# Diversity of the Obligate Gut Bacteria Indicates Host–Symbiont Coevolution at the Population Level in the Plataspid Stinkbug *Megacopta cribraria*

**DOI:** 10.1002/ece3.70611

**Published:** 2024-11-25

**Authors:** Yuan Wang, Xiu‐Xiu Zhu, Bo‐Ren Xiao, Xin‐Rui Hou, Yu‐Xin Liu, Jia‐Yue Zhou, Yi‐Peng Ren, Wen‐Jun Bu, Huai‐Jun Xue

**Affiliations:** ^1^ Institute of Entomology, College of Life Sciences Nankai University Tianjin China

**Keywords:** amplicon sequencing, *beta* diversity, clone, Hemiptera, *Ishikawaella*

## Abstract

*Ishikawaella* is an obligate gut bacterium in stinkbugs that belong to Plataspidae family (Hemiptera: Heteroptera). It is vertically transmitted to newborn nymphs through capsules laid on eggs by maternal stinkbugs. Previous research has established a pattern of strict cospeciation between Plataspidae stinkbugs and *Ishikawaella*. However, the possibility of host–symbiont coevolution at the population level within Plataspidae stinkbugs has not been thoroughly explored. This study analyzed the samples of *Megacopta cribraria* from three phylogenetic clades to investigate host–symbiont coevolution in this insect species. We compared data from third‐generation sequencing (PacBio), next‐generation sequencing (Illumina), and first‐generation sequencing (Sanger), and the results indicated that Illumina sequencing most accurately represents the composition of gut bacterial communities. All stinkbug individuals shared a dominant amplicon sequence variant (ASV), which accounted for an average of 65.99% of *Ishikawaella* sequences (ranging from 58.68% to 87.01%). The top five ASVs (ASV0–ASV4) represented 99.82% of all *Ishikawaella* sequences. Among these, the number of base substitutions between any two ASVs ranged from 1 to 3, significantly lower than the number of substitutions between the main and minor ASVs. This finding suggests that closely related strains are likely to coexist in the same host. Beta diversity analyses revealed significant differences in *Ishikawaella* composition among the three phylogenetic clades, providing evidence for host–symbiont coevolution at the population level in Plataspidae stinkbugs.

## Graphical abstract

Illumina sequencing more accurately reflects the composition of the gut bacterial community in Ishikawaella–Plataspidae system. Closely related strains of Ishikawaella are likely to coexist in the same host. Host–symbiont coevolution exists at the population level of Plataspidae stinkbug Megacopta cribraria.

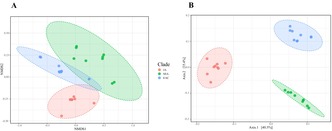

## 1 Introduction

Due to the inability to synthesize essential amino acids and vitamins, insects employ two main strategies to acquire these nutrients. The first is direct ingestion of food, and the second is through the synthesis of symbiotic microbes (Salem et al. 2014). Hemipteran insects, such as stinkbugs (Heteroptera), which have piercing‐sucking mouthparts, primarily consume the phloem or xylem saps, which do not provide sufficient nutrients, leading to nutritional deficiencies in the insects (Moriyama and Fukatsu 2022; Engel and Moran 2013). To address these deficiencies, stinkbugs typically host specific nutritional microbes in their specialized symbiotic organs (Hosokawa et al. 2005; Zytynska, Tighiouart, and Frago 2021; Moriyama and Fukatsu 2022).

The transmission strategies of symbiotic bacteria that provide nutrients to stinkbugs vary significantly among different groups. In *Cimex lectularius* (Cimicidae) and *Nysius* spp. (Lygaeidae), intracellular symbionts are transmitted vertically from the maternal parent to offspring through the ovaries (Hosokawa et al. 2010; Matsuura et al. 2012). By contrast, many species within Pentatomomorpha harbor specific extracellular bacteria in their midgut crypts. However, in most species of the Dinidoridae, Coreoidea, Lygaeoidea, and Pyrrhocoroidea, symbiotic bacteria are not transmitted vertically; instead, they are acquired from the environment through horizontal transmission (Kikuchi, Hosokawa, and Fukatsu 2007, 2011). In some other groups, extracellular bacteria are transmitted vertically from the maternal parent to offspring through specialized mechanisms on their eggs, for example, through surface contamination in Acanthosomatidae, a jelly covering in Urostylidae, and a symbiont capsule in the Plataspidae (Kikuchi et al. 2009; Kaiwa et al. 2014; Fukatsu and Hosokawa 2002; Hosokawa et al. 2006; Hosokawa, Kikuchi, and Fukatsu 2007; Couret et al. 2019; Hosokawa and Fukatsu 2020). In some families, such as Pentatomidae, Scutelleridae, and Blissidae, symbionts may be transmitted both vertically and horizontally through the environmental, incorporating strategies from both transmission modes (Bistolas et al. 2014; Itoh et al. 2014; Duron and Noël 2016; Kashkouli, Fathipour, and Mehrabadi 2019; Hosokawa et al. 2019).

Host–symbiont cospeciation has been observed in the Urostylidae, Acanthosomatidae, and Plataspidae families (Hosokawa et al. 2006; Kikuchi et al. 2009; Kaiwa et al. 2014). Among these, the “plataspid–*Ishikawaella*” system is a well‐studied example of an obligate symbiotic relationship. *Ishikawaella*, a member of the γ‐proteobacteria within the Morganellaceae family, resides extracellularly in numerous crypts within plataspid stinkbugs (Fukatsu and Hosokawa 2002; Hosokawa et al. 2006; Hosokawa, Kikuchi, and Fukatsu 2007). This bacterium has a reduced genome that lacks several genes, including those involved in cell wall synthesis and lipid metabolism. Thus, *Ishikawaella* depends on specific nutrients and metabolic waste from the host's gut for survival and cannot be cultured in vitro (Hosokawa et al. 2006; Nikoh et al. 2011; Couret et al. 2019; Moriyama and Fukatsu 2022). In exchange, I*shikawaella* synthesizes essential amino acids that are crucial for the normal development and reproduction of plataspid stinkbugs (Hosokawa et al. 2006; Moriyama and Fukatsu 2022). To ensure the transfer of *Ishikawaella* between generations, female Plataspidae stinkbugs deposit symbiont capsules on their eggs during oviposition. The newly hatched nymphs acquire *Ishikawaella* by ingesting these capsules from the surface of the egg mass (Fukatsu and Hosokawa 2002; Hosokawa et al. 2006; Hosokawa, Kikuchi, and Fukatsu 2007; Couret et al. 2019; Hosokawa and Fukatsu 2020).

Although cospeciation between hosts and their symbionts has been confirmed in the plataspid–*Ishikawaella* system (Hosokawa et al. 2006), the diversity of *Ishikawaella* within individual plataspid stinkbug species has not been extensively studied. The bean bug, *Megacopta cribraria*, a member of the Plataspidae family, is a common pest in Asia known for its preference for legumes, particularly soybeans and kudzu (Zhu et al. 2023). Previous studies based on mitochondrial gene data (Zhu et al. 2022) and genome resequencing data (Zhu et al. unpublished data) suggested there are three phylogenetic clades within this species, each with a distinct geographical distribution: Southeast Asia (SEA), East Asia continent (EAC), and Japan (JA). In this study, we examined bacterial 16S rRNA genes from 27 *M. cribraria* samples from these clades (nine from each clade) using PacBio, Illumina, and Sanger sequencing techniques to assess their reliability. We mainly used Illumina sequencing data to explore the diversity of *Ishikawaella* based on the comparison of the above sequencing results. We examined the patterns of coexistence among different *Ishikawaella* strains within individual hosts and investigated whether coevolution occurs at the population level in plataspid stinkbugs. This study provides new insights into the nature of symbiosis between stinkbugs and *Ishikawaella*.

## 2 Material and Methods

### 2.1 Sample Collection and DNA Extraction

**FIGURE 1:**
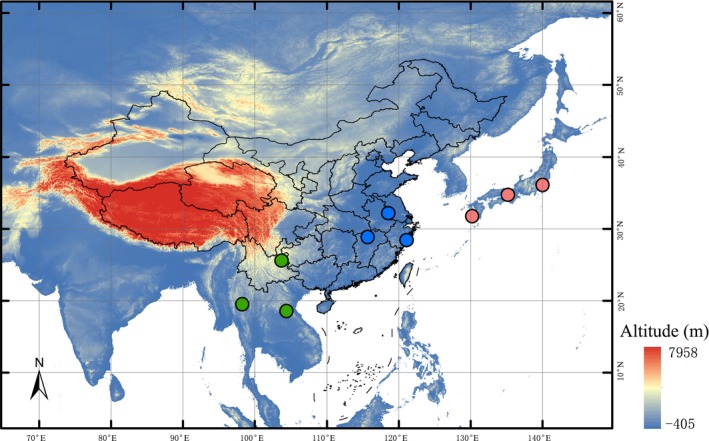
Map illustrating collection locations for the present study. Blue dots: East Asia continent (EAC) clade; green dots: Southeast Asia (SEA) clade; red dots: Japan (JA) clade.

We selected 27 samples of *M. cribraria* from China, Japan, Laos, and Thailand, representing three phylogenetic clades, namely SEA, EAC, and JA (Zhu et al. 2022 and additional unpublished data) (Table 1; Figure 1).

**TABLE 1:** Information of the samples of *Megacopta cribraria*.

Sample ID	Clade	Locality	Geographical coordinates	Collection date
JSNP01	EAC	Pukou, Nanjing, Jiangsu, China	32.11′N, 118.57′E	July 17, 2019
JSNP02	EAC			
JSNP03	EAC			
JXNW01	EAC	Wanli, Nanchang, Jiangxi, China	28.83′N, 115.71′E	July 26, 2019
JXNW02	EAC			
JXNW03	EAC			
ZJWL01	EAC	Leqing, Wenzhou, Zhejiang, China	28.37′N, 121.11′E	July 8, 2018
ZJWL02	EAC			
ZJWL03	EAC			
LAVN01	SEA	Viengthong Bolikhamsai, Laos	18.55′N, 104.44′E	August 21, 2019
LAVN02	SEA			
LAVN03	SEA			
TAMH01	SEA	Mae Hong Son Pangmapha Soppong, Thailand	19.46′N, 98.30′E	August 27, 2018
TAMH02	SEA			
TAMH03	SEA			
YNQQ01	SEA	Sanyan, Qujing, Yunnan, China	25.54′N, 103.72′E	July 16, 2019
YNQQ02	SEA			
YNQQ03	SEA			
JAHK01	JA	Kobe, Hyogo, Japan	34.69′N, 135.19′E	June 13, 2019
JAHK02	JA			
JAHK03	JA			
JAIT01	JA	Tsukuba, Ibaraki, Japan	36.08′N, 140.08′E	June 25, 2019
JAIT02	JA			
JAIT03	JA			
JALK01	JA	Lchiki‐Kushikino, Kagoshima, Japan	31.71′N, 130.27′E	June 15, 2019
JALK02	JA			
JALK03	JA			

Because of the use of alcohol for preservation, the guts of the specimens could not be entirely separated; thus, the entire abdominal contents were dissected for analysis. Genomic DNA was extracted from these samples by using the Universal Genomic DNA Kit (CWBIO, Jiangsu, China), following the manufacturer's instructions. The concentration and purity of DNA were assessed using 1% agarose gel electrophoresis. Based on the concentrations measured, DNA was diluted to 1 ng/μL with sterile water for further analysis.

### 2.2 Sequencing and Data Processing

The same DNA samples were sequenced using the third‐, next‐, and first‐generation sequencing methods to analyze the 16S rRNA genes: Third‐generation sequencing was used to obtain the full‐length sequences of 16S rRNA genes, next‐generation sequencing targeted the V3‐V4 hypervariable regions of the 16S rRNA, and first‐generation sequencing was employed to sequence clones of full‐length 16S rRNA genes.

#### 2.2.1 Third‐Generation Sequencing

The full‐length 16S rRNA gene was amplified from genomic DNA by using the universal primers 27F (5′‐AGRGTTYGATYMTGGCTCAG‐3′) and 1492R (5′‐RGYTACCTTGTTACGACTT‐3′). Libraries for sequencing were prepared using the SMRTbell Template Prep Kit (PacBio) according to the manufacturer's protocols. Library quality was assessed using the Qubit 2.0 Fluorometer (Thermo Scientific) and the FEMTO Pulse system. Sequencing was performed on the PacBio Sequel II platform at Novogene Biotech Co., Ltd.

Raw sequences were initially processed using the PacBio SMRT portal, where reads were assigned to samples based on unique barcodes and trimmed to remove the barcode and primer sequences. The resulting circular consensus sequencing (CCS) reads were quality‐controlled and chimera‐filtered using the DADA2 package (v. 1.18) (Callahan et al. 2016), generating amplicon sequence variant (ASV) tables and representative sequences. These outputs were then imported into QIIME2 (v. 2021.4) (Bolyen et al. 2019) for further analysis. ASVs containing fewer than five sequences were filtered out using the q2‐feature‐table plugin. Representative sequences were assigned taxonomies using the q2‐feature‐classifier plugin with a pre‐fitted sklearn‐based taxonomy classifier method (Bokulich et al. 2018). The SILVA database (https://www.arb‐silva.de/) was used to annotate the ASV tables for bacterial identification (Wang et al. 2007; Quast et al. 2013).

#### 2.2.2 Next‐Generation Sequencing

The primers 341F (5′‐CCTACGGGAGGCAGCAG‐3′) and 806R (5′‐GGACTACHVGGGTWTCTAAT‐3′) were used to amplify the V3‐V4 regions of the 16S rRNA gene. The resulting PCR products were combined in equal proportions and purified using the Qiagen Gel Extraction Kit (Qiagen, Germany) according to the manufacturer's instructions. Sequencing libraries were then prepared using the NEBNext Ultra II DNA Library Prep Kit (Cat No. E7645), and the quality of these libraries was assessed using the Qubit 2.0 Fluorometer (Thermo Scientific) and the Agilent Bioanalyzer 2100 system. The libraries were sequenced on an Illumina NovaSeq platform by Novogene Biotech Co. Ltd. producing 250 bp paired‐end reads.

These paired‐end reads were assigned to samples based on unique barcodes and truncated to remove barcode and primer sequences. The reads were then merged using FLASH (v. 1.2.11, http://ccb.jhu.edu/software/FLASH/) (Magoč and Salzberg 2011). Quality filtering was conducted using fastp (v. 0.20.0) to generate high‐quality clean tags. These clean tags were compared against the SILVA database using VSEARCH (v. 2.15.0) to identify and remove chimera sequences, resulting in effective tags (Haas et al. 2011). Denoising was performed using DADA2 within QIIME2 (Version QIIME2‐202006) to produce initial ASVs, filtering out ASVs with abundances < 5. Species annotation was also performed using QIIME2, utilizing the SILVA database (https://www.arb‐ silv.de/for16s) for accurate 16S rRNA gene annotation (Bolyen et al. 2019).

#### 2.2.3 Cloning and First‐Generation Sequencing

The full‐length 16S rRNA genes of the bacteria were cloned from nine representative samples, with three samples from each of the identified clades. Cloning was conducted using the standard protocols of the pLB‐T Fast Ligation Kit (VT217) (Tiangen Biotech, Beijing, China). PCR products were generated using the primers 16SA1 (5′‐AGAGTTTGATCMTGGCTCAG‐3′) and 16SB1 (5′‐TACGGYTACCTTGTTACGACTT‐3′). These products were then purified using the TaKaRa MiniBEST Agarose Gel DNA Extraction Kit Version 4.0 (Takara Biomedical Technology, Beijing, China) and were subsequently inserted into the pLB‐T vector (TIANGEN BIOTEC, Beijing, China). The resulting vectors were transformed into *Escherichia coli* DH5α component cells according to the manufacturer's instructions. Transformants were selected on LB plates containing ampicillin. Plasmids were directly sequenced using the pLB forward (5′‐CGACTCACTATAGGGAGAGCGGC‐3′) and pLB reverse (5′‐AAGAACATCGATTTTCCATGGCAG‐3′) primers provided in the kit. More than 30 randomly selected clones from each of the nine representative samples were sequenced using the ABI3700 sequencer at BGI Genomics Co. Ltd. The sequences of the clones were then identified by BLAST in the NCBI.

### 2.3 Strain‐Level Diversity of *Ishikawaella*

The absolute and relative contents of *Ishikawaella* in each sample and the relative contents of each ASV of *Ishikawaella* were statistically analyzed. Notably, one ASV defined on the basis of V3‐V4 segments might correspond to multiple ASVs defined on the basis of the full‐length 16S rRNA gene. To compare data from third‐ and first‐generation sequencing with those from second‐generation sequencing, which involves shorter sequences, we truncated the sequences from the first two methods to match the length of the second‐generation sequences. After this adjustment, we recalculated the data to evaluate the consistency of *Ishikawaella* ASVs detected across the three sequencing technologies.

### 2.4 Phylogenetic Relationships

To assess genetic differentiation among *Ishikawaella* ASVs from next‐generation sequencing data, we computed the pairwise genetic distances of the 41 ASVs by using MEGAX software (v. 7.0.14) (Kumar, Stecher, and Tamura 2016). In addition, we analyzed the relationship between *Ishikawaella* ASVs identified in *M. cribraria* by summarizing the distribution of 16S V3‐V4 haplotypes by using statistical parsimony networks, inferred with TCS software (v. 1.2.1) (Clement, Posada, and Crandall 2000). For the phylogenetic analysis of *Ishikawaella* strains obtained in this study, we used 16S V3‐V4 sequences from next‐generation sequencing combined with 12 *Ishikawaella* 16S sequences downloaded from the NCBI. The sequences were aligned using MEGAX software (Kumar, Stecher, and Tamura 2016). Neighbor‐joining (NJ) trees were then constructed using MEGAX on the basis of the Kimura 2‐parameter (K2P) distance model. Bootstrap analysis with 1000 replications was performed to evaluate the support for each branch in the NJ trees. Maximum likelihood (ML) trees were also reconstructed using RAxML (v. 8.2.9) (Stamatakis 2006) on the CIPRES Science Gateway (v. 3.3) (Miller, Pfeiffer, and Schwartz 2010), with 1000 nonparametric bootstrap replicates to ensure reliability. The trees were rooted post hoc by using *Buchnera* sp. (accession number: M27039) as an outgroup. The resulting phylogenetic trees were visualized and edited using FigTree (v. 1.4.4) (Rambaut 2018) and Adobe Illustrator CS5.

### 2.5 *Alpha* and *Beta* Diversity of *Ishikawaella*

To achieve a consistent sequencing depth across all samples, we rarefied the data to the smallest library size prior to conducting alpha and beta diversity analyses. We calculated four microbial alpha diversity indices for each sample: Chao1, ACE, Simpson, and Shannon. We used the nonparametric Kruskal–Wallis test to determine whether differences in these diversity indices among the different host clades were significant. For beta diversity analysis, we employed a combination of two ordination methods—nonmetric multidimensional scaling (NMDS) and principal coordinates analysis (PCoA)—along with two distance metrics, Jaccard and Bray–Curtis. We also applied two statistical methods, permutational multivariate analysis of variance (PerMANOVA), and analysis of similarities (ANOSIM), to evaluate whether the composition of *Ishikawaella* differed significantly across host clades. All analyses were performed using the MicrobiomeAnalyst platform (https://www.microbiomeanalyst.ca/) (Dhariwal et al. 2017; Chong et al. 2020).

## 3 Results

### 3.1 Overall Bacterial Diversity

#### 3.1.1 Third‐Generation Sequencing

**FIGURE 2:**
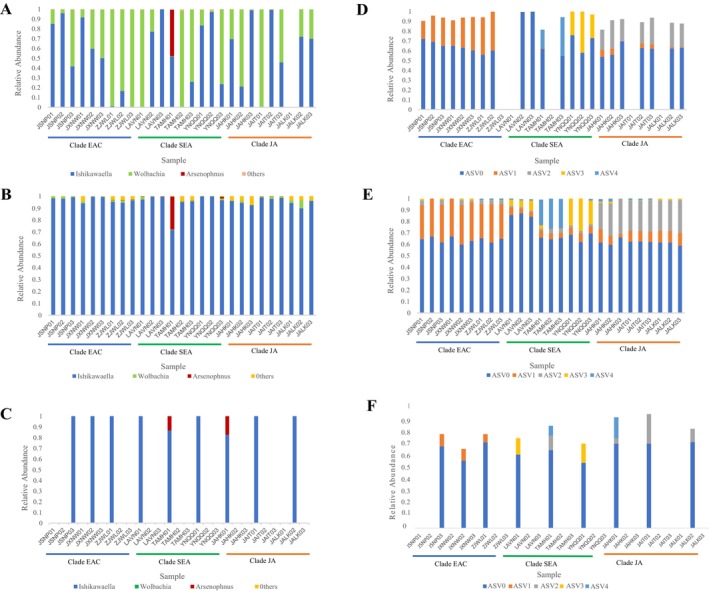
Diversity of the gut bacteria in *Megacopta cribraria*. (A–C) Proportions of *Ishikawaella*, *Wolbachia*, *Arsenophnus*, and other bacterial sequences in the total bacterial sequences, as estimated using third‐, next‐, and first‐generation sequencing data. (D–F) Relative abundance of major *Ishikawaella* amplicon sequence variants (ASVs), as estimated using third‐, next‐, and first‐generation sequencing data.

One of the samples (JALK01) could not be sequenced successfully. In the remaining 26 samples, we obtained a total of 262,731 reads, averaging 10,105 reads per sample with a standard deviation of ±2014, ranging from 7048 to 14,643 reads. From these reads, we identified a total of 370 bacterial ASVs. Among these, 170 ASVs corresponding to 138,156 reads were assigned to the genus *Ishikawaella*, present in 24 samples and accounting for 53.01% (ranging from 0% to 99.94%) of the bacterial sequences. In addition, 112 ASVs with 120,149 reads were assigned to *Wolbachia*, found in 25 samples and accounting for 44.96% (ranging from 0% to 100%) of the total bacterial sequences. Finally, 52 ASVs corresponding to 4093 reads were identified as *Arsenophonus*, present in one sample and accounting for 1.56% of the sequences. Collectively, sequences from these three genera constituted 99.87% of all bacterial sequences analyzed (Figure 2A).

#### 3.1.2 Next‐Generation Sequencing

We obtained a total of 1,777,722 reads from 27 samples, with an average of 65,842 reads per sample, and a standard deviation of ±5071 (ranging from 49,237 to 74,412 reads). A total of 605 bacterial ASVs were identified. Of these, 42 ASVs, comprising 1,708,966 reads, were assigned to *Ishikawaella* and found in all 27 samples. These accounted for 95.87% of the bacterial sequences, with individual sample proportions ranging from 71.66% to 99.85%. Additionally, four ASVs with 21,066 reads were assigned to *Wolbachia* and detected in all 27 samples and accounting for 1.18% of the bacterial sequences (ranging from 0.08% to 6.76%). Nine ASVs with 15,808 reads were identified as *Arsenophonus*, found in 14 samples and accounting for 1.15% of the bacteria, with proportions ranging from 0% to 27.62%. Notably, an unusually high proportion of 27.62% was detected in one sample (TAMH01). Collectively, these three genera accounted for 98.21% of the total bacterial sequences (Figure 2B).

#### 3.1.3 First‐Generation Sequencing

A total of 275 clones from nine representative samples were successfully sequenced. Among these, 266 clones identified as belonging to *Ishikawaella* were found across all nine samples, whereas nine clones belonging to *Arsenophonus* were detected in only two samples (Figure 2C).

### 3.2 Strain‐Level Diversity of *Ishikawaella*

Although third‐generation sequencing results indicated that *Ishikawaella* accounted for more than 50% of the bacterial sequences, there were inconsistencies in five individuals where I*shikawaella* was either not detected or represented only a very small proportion of the bacterial sequences (Figure 2A). This finding is counterintuitive and inconsistent with the findings of previous studies.

Based on next‐generation sequencing, all 27 samples yielded high read counts for *Ishikawaella*, with an average of 63,295 reads per sample (ranging from 35,284 to 74,074) (Table S1). We detected a total of 42 *Ishikawaella* ASVs. The diversity within the *Ishikawaella* communities in *M. cribraria* was relatively low, with the number of ASVs per sample ranging from 3 to 12 (Table S1). The five most prevalent ASVs (ASV0–ASV4) accounted for 99.82% of all *Ishikawaella* sequences (Figure 2E). All samples shared the most abundant ASV (ASV0), which, on average, accounted for 65.99% (ranging from 58.68% to 87.01%) of the *Ishikawaella* sequences (Figure 2E). However, the second most abundant ASV varied across different host clades: ASV1 was predominant in the EAC clade, ASV2 in the JA clade, and ASV3 or ASV4 in the SEA clade (Figure 2E). When adjusted for comparison, the proportion of ASV0 was the highest in both third‐generation and first‐generation sequencing data. Furthermore, the distribution of the second most abundant ASV in each sample aligned closely with the findings of the next‐generation sequencing data (Figure 2D,F). For third‐generation sequencing, among the 22 samples containing high *Ishikawaella* content, an average of 93.23% of truncated reads (430 bp) were identified to the five major ASVs (ASV0–ASV4) obtained by next‐generation sequencing (Figure 2D). For first‐generation sequencing, 75.6% (201 out of 266) truncated clones (430 bp) were identified as belonging to the five main ASVs (ASV0–ASV4) generated from next‐generation sequencing (Figure 2F).

### 3.3 Phylogenetic Analysis

**FIGURE 3:**
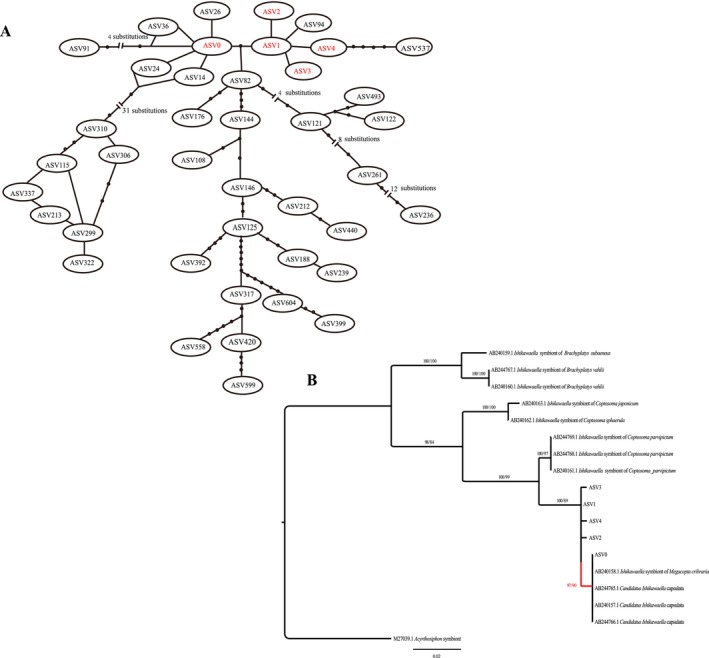
Phylogenetic analysis of *Ishikawaella* strains from *M. cribraria* . (A) Maximum parsimony network for the V3‐V4 hypervariable region of the16S rRNA from 41 *Ishikawaella* amplicon sequence variants (ASVs) based on Illumina sequencing. Straight lines and small dots reflect mutations and median vectors, respectively. (B) Maximum likelihood (ML) tree of *Ishikawaella* reconstructed using RaxML based on the V3‐V4 hypervariable region of 16S rRNA. The tree was constructed using five major *Ishikawaella* ASVs from *M. cribraria* obtained in the present study and 12 *Ishikawaella* sequences downloaded from NCBI, with *Buchnera* (M27039) as an outgroup. The values above the branches represent ML (first number) and NJ (second number) bootstrap support values (> 50% are shown).

Among the 41 *Ishikawaella* ASVs analyzed (the ASV505, with a length of 247 bp, was excluded from the analysis due to its significantly shorter size compared to the amplicon length of 430 bp utilized in this study), the number of base substitutions between any two ASVs ranged from 1 to 39 (Table S2). The parsimony network for the 16S rRNA genes revealed that the number of nucleotide base substitutions between each of the five major ASVs (ASV0–ASV4) ranged from 1 to 3 (Figure 3A). Both the ML and NJ trees displayed similar topologies, indicating that the 41 ASVs identified from the next‐generation sequencing data in this study, along with the four *Ishikawaella* sequences from *M. cribraria* reported previously by Hosokawa et al. (2006), clustered into a single clade (Figure S1). Further phylogenetic analysis of the five main ASVs showed that ASV0, which had the highest sequence proportion, was the closest to the four sequences reported by Hosokawa et al. (2006) (Figure 3B).

### 3.4 *Alpha* and *Beta* Diversity of *Ishikawaella*

**FIGURE 4:**
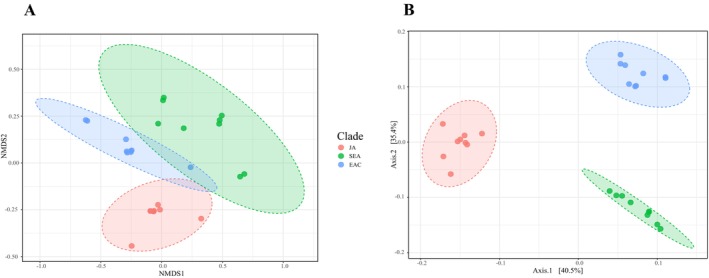
Nonmetric multidimensional scaling (A) and principal coordinate analysis plots (B) visualizing *Ishikawaella* community dissimilarities of three clades of *M. cribraria* using the Bray–Curtis distance methods.

All the rarefaction curves plateaued, demonstrating that the sequencing depth was sufficient for the analysis (Figure S2). Significant differences were observed in three of the *alpha* diversity indices: Chao1 (H = 6.8738, *p* = 0.032165), Shannon (H = 11.143, *p* = 0.003805), and Simpson (H = 12.074, *p* = 0.0023886); however, no significant difference was found in ACE (H = 0.063131, *p* = 0.96893) (Figure S3). Both ANOSIM and PerMANOVA tests indicated significant differences in the *beta* diversity of *Ishikawaella* across the three host clades (ANOSIM: Jaccard, *R* = 0.87266, *p* < 0.001; Bray–Curtis, *R* = 0.87266, *p* < 0.010. PerMANOVA: Jaccard, *R* ^2^ = 0.71531, *p* = 0.001; Bray–Curtis, *R* ^2^ = 0.73592, *p* = 0.001) (Figure 4; Figure S4).

## 4 Discussion

In the present study, we analyzed the diversity of *Ishikawaella* harbored by *M. cribraria* to investigate host–symbiont coevolution at the population level in Plataspidae stinkbugs based on data from third‐, next‐, and first‐generation sequencing. Although the third‐generation sequencing results showed that *Ishikawaella* constituted more than 50% of the bacterial population, it was either undetected or present in very low proportions in 5 out of 26 individuals (Figure 2A). This finding is inconsistent with those of previous studies based on clonal sequencing and whole‐genome shotgun sequencing, which found that *Ishikawaella* dominated the gut bacteria of Plataspidae stinkbugs (Hosokawa et al. 2006; Nikoh et al. 2011). To verify the accuracy of the third‐generation sequencing results and better understand the diversity of *Ishikawaella*, both next‐ and first‐generation sequencing were subsequently performed on the same DNA samples.

The results obtained by both next‐generation sequencing and first‐generation sequencing support the significant dominance of *Ishikawaella* in the bacterial community (Figure 2A–C). The first‐generation sequencing data revealed greater consistency with the next‐generation sequencing results than with the third‐generation data (Figure 2A–C). This finding suggests that third‐generation sequencing may have overestimated the presence of *Wolbachia* and underestimated *Ishikawaella* abundance. Compared with next‐generation sequencing, the small sample size sampling method of cloning sequencing may make it difficult to detect the ASVs with low abundance, or lead to an imbalance in the proportions of different ASVs. Thus, we inferred that the next‐generation sequencing data likely provide a more accurate reflection of the bacterial community structure. Moreover, in samples where all three types of data were successfully obtained, the proportions of the main *Ishikawaella* strains were broadly consistent across the three sequencing methods: ASV0 accounted for the highest proportion among all samples. The second dominant ASVs in most samples of the EAC clade, SEA clade, and JA clade are ASV1, ASV3 (or ASV4), and ASV2, respectively (Figure 2D–F).

Illumina‐based amplicon sequencing of the 16S rRNA gene is widely regarded as the standard method for studying microbial diversity due to its high reliability and low cost (Souza et al. 2023). However, the shorter read lengths of Illumina sequencing might not provide sufficient information for accurate bacterial classification. By contrast, third‐generation sequencing technologies, such as PacBio and Nanopore, generate full‐length 16S rRNA gene reads, offering higher taxonomic resolution (Yang et al. 2021; Yu et al. 2022; Souza et al. 2023; Buetas et al. 2024). Comparative studies have identified biases in both next‐ and third‐generation amplicon sequencing, which vary in their nature and impact. These discrepancies can arise from factors such as the specificity of primers, stringency of PCR conditions, and differences in read length and coverage (Wagner et al. 2016; Katiraei et al. 2022; Khalaf et al. 2023; Buetas et al. 2024). Third‐generation sequencing results are not always superior to those from next‐generation sequencing. For instance, although third‐generation sequencing platforms can detect many genera of the nasal microbiota similar to Illumina, they could not detect certain taxa, such as the genus *Corynebacterium* (Heikema et al. 2020). By comparing data from three sequencing platforms, our study provides robust evidence supporting these observations. As we mentioned, third‐generation sequencing may have overestimated the abundance of *Wolbachia* and underestimated the abundance of *Ishikawaella* in this study; however, we cannot be sure of how the sequencing will behave with different symbiont assemblages—that is, which ones will be underestimated and which ones will be overestimated. Therefore, it is more reliable to use multiple sequencing methods to assess which is closer to the real situation, and the most suitable technique should be evaluated case by case, rather than giving a general rule. Long‐read sequencing may be not necessary if one is looking for a general overview of the bacterial diversity; instead, it should be preferred to explore the diversity of a dominant symbiont, as demonstrated by the higher number of ASVs related to *Ishikawaella* in third‐generation sequencing.

Phylogenetic analysis indicated that ASV0 was most closely related to the strains (AB240158.1, AB244765.1, AB240157.1, and AB244766.1) previously identified in *M. cribraria* (Figure 3B). This close relationship is likely because only a small number of clones were selected for sequencing from each midgut DNA sample in the previous study, leading to the detection of the most abundant strains (Fukatsu and Hosokawa 2002; Hosokawa et al. 2006).

Our study also demonstrated that the diversity of *Ishikawaella* harbored by *M. cribraria* was relatively low, with only 3–12 ASVs and comprising 2–5 main ASVs in each sample. Furthermore, the number of nucleotide base substitutions between the five major ASVs (ASV0–ASV4) was small, ranging from 1 to 3, which was significantly lower than the substitutions observed between the main and minor ASVs (Figure 3A; Table S2). A newborn nymph of *M. cribraria* acquires approximately 2 × 10^7^ symbionts from the symbiont capsule, and the minimum threshold for successful vertical transmission was estimated to be 1.9 × 10^6^ symbionts (Hosokawa et al. 2007). Over time, these symbionts establish a large population in the midgut crypts, which then becomes a hotspot for genetic mutations. Symbiotic specificity may be affected by microbe–microbial competition at the species level (Itoh et al. 2019; Andongma et al. 2022). We hypothesize that the competition between *Ishikawaella* strains might facilitate the purging of mutations, making it challenging for strains that are genetically distant from the main strains to persist amid competition. Furthermore, we speculate that fewer mutations at nucleotide sites do not significantly alter the physiological characteristics of the bacteria, allowing such strains to coexist more easily with the original strain. By contrast, strains with more mutations might be outcompeted by the “original strains,” which have long adapted to the host environment. Thus, several closely related major ASVs continue to coexist in a significant proportion of the host population and can be transmitted vertically to subsequent generations. However, since the study is only based on a 16S rRNA fragment, it is not clear how exactly would these mutations relate to the fitness/competitiveness of the strains. Here, the mutation number in this marker (16S rRNA) is only considered as a proxy for the overall mutational load of the genome. Further work is needed to verify the hypothesis stated above.

All host individuals across three clades, spanning a large geographic area, share the same predominant ASV of *Ishikawaella* (Figure 2E). However, the second largest ASV varied depending on the region and the host: ASV1 in EAC clade, ASV3 or ASV4 in SEA clade, and ASV2 in JA clade (Figure 2E), suggesting a pattern of coevolution between the host and symbiont at the population level in the plataspid stinkbug. This pattern is further supported by the results of the beta diversity analysis of *Ishikawaella* (Figure 4).

Overall, this study suggests that closely related strains of *Ishikawaella* are more likely to coexist in the same host and provides evidence for host–symbiont coevolution at the population level in Plataspidae stinkbugs, enriching our understanding of the nature of symbiosis between stinkbugs and *Ishikawaella*.

## Author Contributions

**Yuan Wang:** data curation (equal), formal analysis (equal), investigation (equal), writing – original draft (equal). **Xiu‐Xiu Zhu:** data curation (equal), formal analysis (equal), writing – review and editing (equal). **Bo‐Ren Xiao:** formal analysis (equal), writing – review and editing (equal). **Xin‐Rui Hou:** formal analysis (equal), investigation (equal), methodology (equal), writing – review and editing (equal). **Yu‐Xin Liu:** formal analysis (equal), investigation (equal), methodology (equal). **Jia‐Yue Zhou:** formal analysis (equal), writing – review and editing (equal). **Yi‐Peng Ren:** methodology (equal), writing – review and editing (equal). **Wen‐Jun Bu:** conceptualization (equal), writing – review and editing (equal). **Huai‐Jun Xue:** conceptualization (equal), funding acquisition (equal), project administration (equal), supervision (equal), writing – review and editing (equal).

## Conflicts of Interest

The authors declare no conflicts of interest.

## Supporting information


**Figure S1.** Maximum likelihood (ML) tree of *Ishikawaella* reconstructed using RaxML based on the V3‐V4 hypervariable region of 16S rRNA. The tree was constructed using 41 *Ishikawaella* ASVs from *M. cribraria* obtained in the present study, and 12 *Ishikawaella* sequences downloaded from the NCBI, with *Buchnera* (M27039) as an outgroup. The values above the branches represent ML (first number) and NJ (second number) bootstrap support values (> 50% are shown).
**Figure S2.** Rarefaction curves for *Ishikawaella* in the *Megacopta cribraria* samples.
**Figure S3.** Alpha diversity of symbiotic *Ishikawaella* communities, including Shannon index, Chao1 index, Simpson index, and ACE index in *Megacopta cribraria* samples.
**Figure S4.** Nonmetric multidimensional scaling (A) and principal coordinate analysis plots (B) visualizing *Ishikawaella* community dissimilarities of the three clades of *M. cribraria* using Jaccard distance methods.

**Table S1.** Feature table of Ishikawaella.

**Table S2.** Distance between each pair of Ishikawaella ASVs generated by next‐generation sequencing.

## Data Availability

Raw reads were deposited at NCBI within the BioProject ID PRJNA1132412 (third‐generation sequencing data), PRJNA1132485 (next‐generation sequencing data), and PRJNA1132377 (first‐generation sequencing data).
