# Supplementary figures and images for: Diversity of the Obligate Gut Bacteria Indicates Host–Symbiont Coevolution at the Population Level in the Plataspid Stinkbug Megacopta cribraria
Source: Ecol Evol. 2024 Nov 25;14(11):e70611. doi: 10.1002/ece3.70611 (PMC11586681; doi:10.1002/ece3.70611)

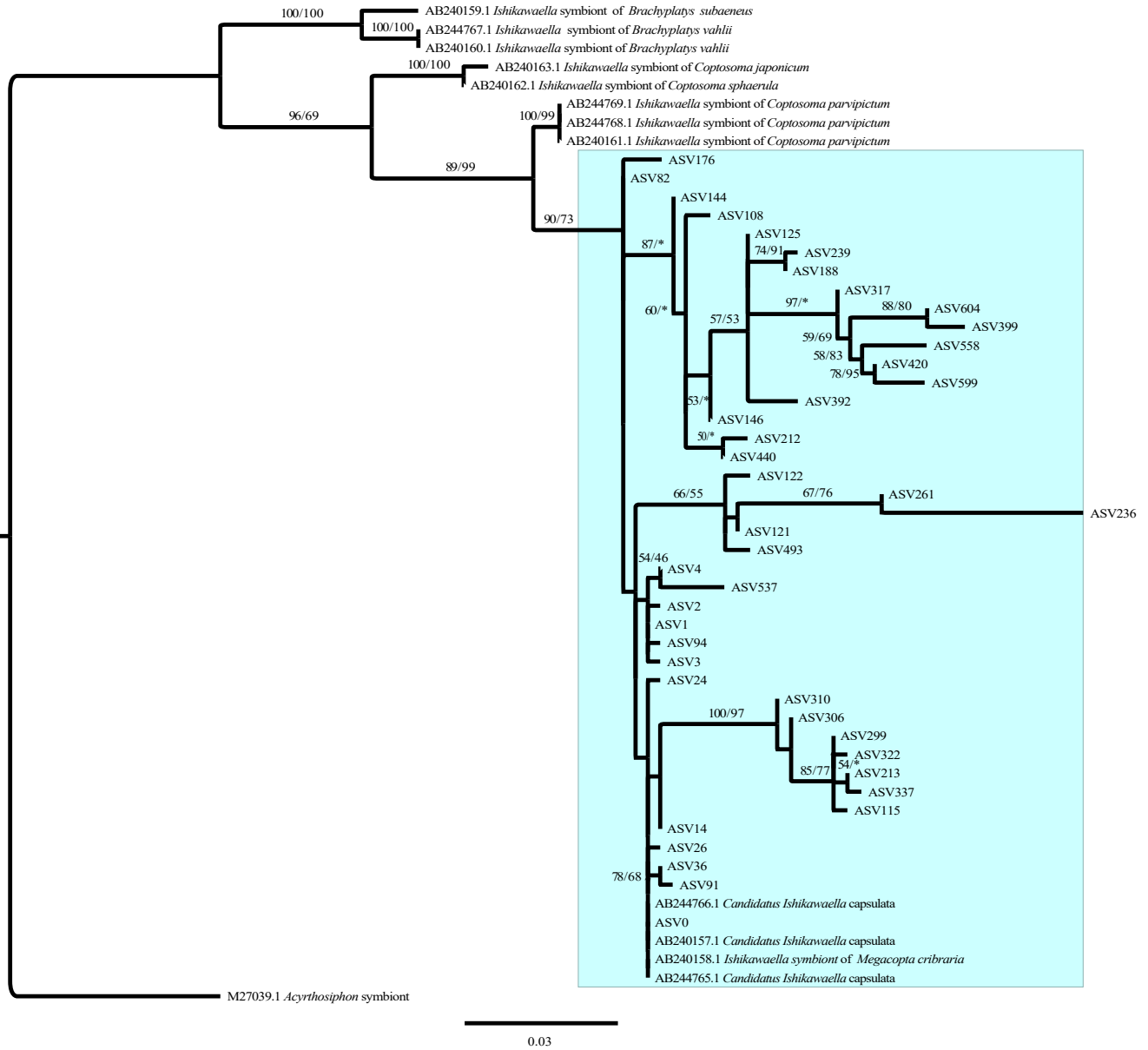

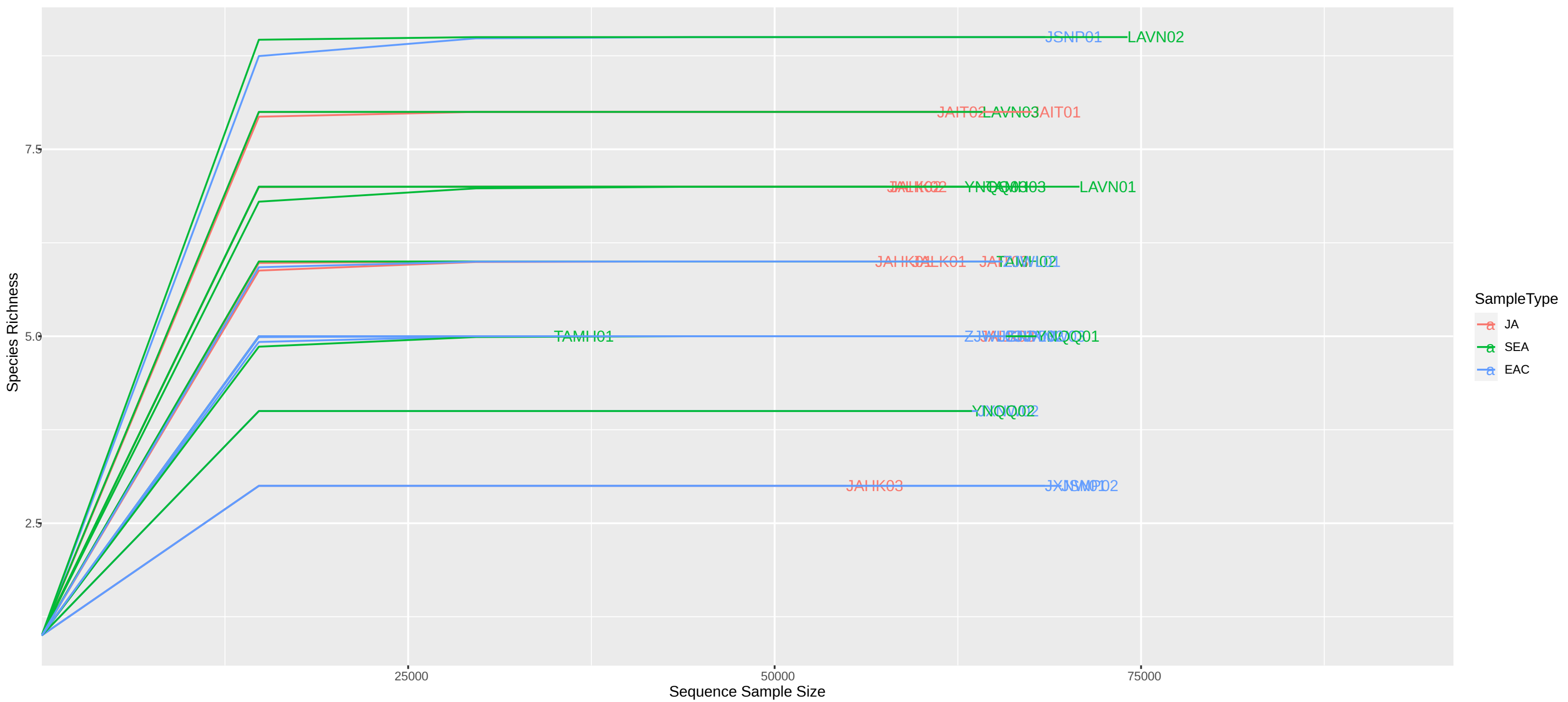

**A**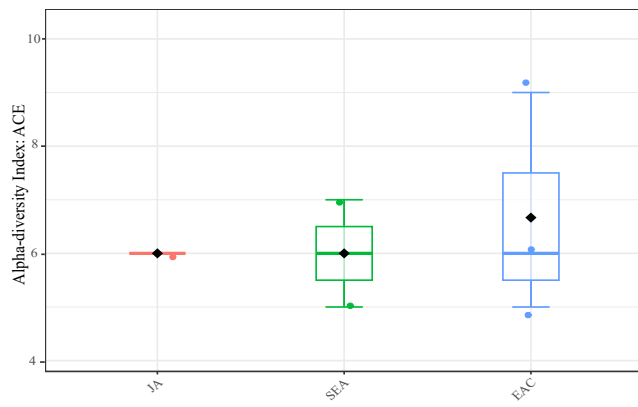**B**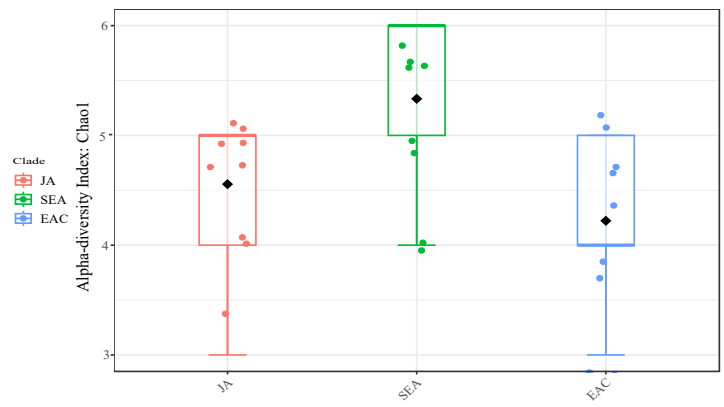**C**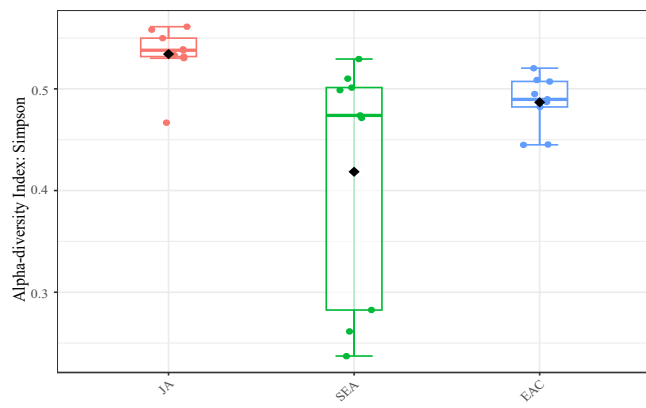**D**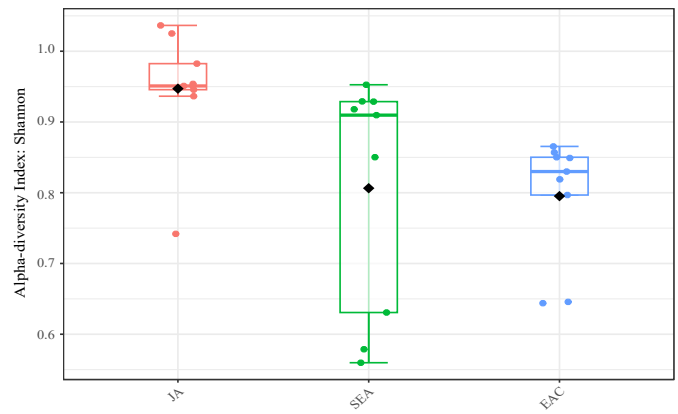

**A**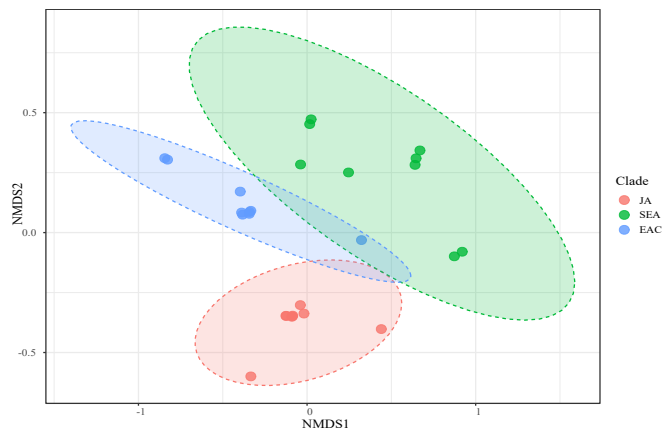**B**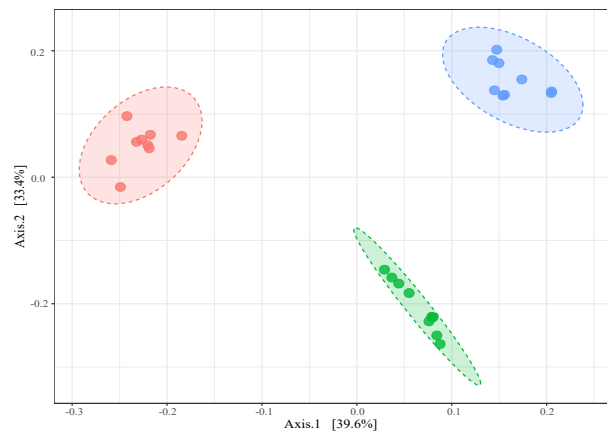

Supplement: Supplementary file 1 — Figure S1. Maximum likelihood (ML) tree of Ishikawaella reconstructed using RaxML based on the V3‐V4 hypervariable region of 16S rRNA. The tree was constructed using 41 Ishikawaella ASVs from M. cribraria obtained in the present study, and 12 Ishikawaella sequences downloaded from the NCBI, with Buchnera (M27039) as an outgroup. The values above the branches represent ML (first number) and NJ (second number) bootstrap support values (> 50% are shown). Figure S2. Rarefaction curves for Ishikawaella in the Megacopta cribraria samples. Figure S3. Alpha diversity of symbiotic Ishikawaella communities, including Shannon index, Chao1 index, Simpson index, and ACE index in Megacopta cribraria samples. Figure S4. Nonmetric multidimensional scaling (A) and principal coordinate analysis plots (B) visualizing Ishikawaella community dissimilarities of the three clades of M. cribraria using Jaccard distance methods. [file ECE3-14-e70611-s002.pdf]
